# Somatostatin-receptor scintigraphy for staging and follow-up of patients with extraintestinal marginal zone B-cell lymphoma of the mucosa associated lymphoid tissue (MALT)-type

**DOI:** 10.1054/bjoc.2001.2070

**Published:** 2001-11

**Authors:** M Raderer, T Traub, M Formanek, I Virgolini, C Österreicher, W Fiebiger, M Penz, U Jäger, J Pont, A Chott, A Kurtaran

**Affiliations:** Departments of Internal Medicine I, Departments of Internal Medicine IV, Nuclear Medicine, Otorhinolaryngology, Clinical Pathology, University of Vienna; Internal Medicine V, Kaiser Franz Josef Spital; Nuclear Medicine, KH Lainz, Vienna, Austria

**Keywords:** somatostatin, extraintestinal MALT-lymphoma, scintigraphy

## Abstract

The majority of lymphomas of the mucosa-associated lymphoid tissue (MALT)-type arise in the stomach, but extragastric locations are also frequently encountered. Due to previous results indicating that somatostatin receptor (SSTR)-expression distinguishes between gastric and extragastric MALT-type lymphoma, we have initiated a study to evaluate the role of SSTR-scintigraphy for staging and follow-up of patients with extragastric manifestations of MALT-type lymphoma. A total of 30 consecutive patients, including 24 with primary extragastric MALT-type lymphoma, 5 patients with dissemination to extragastric sites (including colon, lung, parotid, ocular adnexa and breast) following an initial gastric MALT-lymphoma and one patient with spread to stomach, lung and lymph nodes following parotid lymphoma were prospectively studied. All patients had histologically verified MALT-type lymphoma: 2 patients had lymphoma presenting in the lung, 9 in the ocular adnexa, 7 had lymphomas in the parotid, 2 patients had disease located in the breast, 3 patients had lymph-node relapse following MALT-type lymphoma of the parotid, the lacrimal gland and the thyroid, and 1 had primary MALT-lymphoma of the liver. All patients underwent SSTR-scintigraphy using ^111^In-DTPA-D-Phe^1^-Octreotide (^111^In-OCT) before initiation of therapy, while 13 also had a second scan after treatment. The results of gamma camera imaging were compared to conventional staging. No positive scans could be obtained in patients with dissemination following gastric lymphoma, while all patients with primary extragastric lymphoma had positive scans at the site of histologically documented involvement before initiation of therapy. In addition, also the patient with secondary spread to stomach, lung and lymph nodes was positive in all documented lymphoma sites. In one patient, focal tracer uptake in projection to the maxillary sinus was documented, which was bioptically verified as inflammation. In the scans performed after therapy, focal tracer accumulation in the left orbit indicated persistance of disease following irradiation in one patient with otherwise negative work-up, which was verified by MRI and biopsy 6 months later. In another patient, a positive scan indicated disease relapse in the lacrimal gland 9 months before clinical verification by means of ultrasound. In one patient, a focus not present in the pretherapeutic scan was found in the ethmoidal sinus, corresponding to a hyperplastic polyp. Both SST-scan as well as CT indicated disease persistance in one case, while negative scans corresponding to complete remission as judged by conventional staging were obtained following therapy in the remaining patients, and absence of relapse has been confirmed for a median follow-up of 2 years. These results indicate that ^111^In-OCT is an excellent tool for staging and non-invasive therapy-monitoring in extragastric MALT-type lymphomas. These data further confirm our initial finding that gastric MALT-type lymphomas do not express relevant amounts of respective SSTR, and that SSTR-scanning is able to distinguish between gastric vs extragastric origin of MALT-type lymphoma irrespective of the site of presentation.© 2001 Cancer Research Campaign  http://www.bjcancer.com


British Journal of Cancer (2001) 85(10), 1462–1466
© 2001 Cancer Research Campaign
doi: 10.1054/ bjoc.2001.2070, available online at http://www.idealibrary.com on IDEAL

http://www.bjcancer.com

### Somatostatin-receptor scintigraphy for staging and follow-up of patients with extraintestinal marginal zone B-cell lymphoma of the mucosa associated lymphoid tissue (MALT)-type

**M Raderer[1], T Traub[2], M Formanek[3], I Virgolini[7], C Österreicher[4], W Fiebiger[1], M Penz[1], U Jäger[1], J Pont[6], A Chott[5] and A Kurtaran[2]**

Departments of Internal Medicine I[1] and IV[4], Nuclear Medicine[2], Otorhinolaryngology[3], and Clinical Pathology[5], University of Vienna, Internal Medicine V[6], Kaiser Franz Josef Spital, and Nuclear Medicine, KH Lainz[7], Vienna, Austria

**Summary** The majority of lymphomas of the mucosa-associated lymphoid tissue (MALT)-type arise in the stomach, but extragastric locations are also frequently encountered. Due to previous results indicating that somatostatin receptor (SSTR)-expression distinguishes between gastric and extragastric MALT-type lymphoma, we have initiated a study to evaluate the role of SSTR-scintigraphy for staging and follow-up of patients with extragastric manifestations of MALT-type lymphoma. A total of 30 consecutive patients, including 24 with primary extragastric MALT-type lymphoma, 5 patients with dissemination to extragastric sites (including colon, lung, parotid, ocular adnexa and breast) following an initial gastric MALT-lymphoma and one patient with spread to stomach, lung and lymph nodes following parotid lymphoma were prospectively studied. All patients had histologically verified MALT-type lymphoma: 2 patients had lymphoma presenting in the lung, 9 in the ocular adnexa, 7 had lymphomas in the parotid, 2 patients had disease located in the breast, 3 patients had lymph-node relapse following MALT-type lymphoma of the parotid, the lacrimal gland and the thyroid, and 1 had primary MALT-lymphoma of the liver. All patients underwent SSTR-scintigraphy using $^{111}$In-DTPA-D-Phe$^{1}$-Octreotide ($^{111}$In-OCT) before initiation of therapy, while 13 also had a second scan after treatment. The results of gamma camera imaging were compared to conventional staging. No positive scans could be obtained in patients with dissemination following gastric lymphoma, while all patients with primary extragastric lymphoma had positive scans at the site of histologically documented involvement before initiation of therapy. In addition, also the patient with secondary spread to stomach, lung and lymph nodes was positive in all documented lymphoma sites. In one patient, focal tracer uptake in projection to the maxillary sinus was documented, which was bioptically verified as inflammation. In the scans performed after therapy, focal tracer accumulation in the left orbit indicated persistance of disease following irradiation in one patient with otherwise negative work-up, which was verified by MRI and biopsy 6 months later. In another patient, a positive scan indicated disease relapse in the lacrimal gland 9 months before clinical verification by means of ultrasound. In one patient, a focus not present in the pretherapeutic scan was found in the ethmoidal sinus, corresponding to a hyperplastic polyp. Both SST-scan as well as CT indicated disease persistance in one case, while negative scans corresponding to complete remission as judged by conventional staging were obtained following therapy in the remaining patients, and absence of relapse has been confirmed for a median follow-up of 2 years. These results indicate that $^{111}$In-OCT is an excellent tool for staging and non-invasive therapy-monitoring in extragastric MALT-type lymphomas. These data further confirm our initial finding that gastric MALT-type lymphomas do not express relevant amounts of respective SSTR, and that SSTR-scanning is able to distinguish between gastric vs extragastric origin of MALT-type lymphoma irrespective of the site of presentation. © 2001 Cancer Research Campaign http://www.bjcancer.com

**Keywords**: somatostatin; extraintestinal MALT-lymphoma; scintigraphy

Lymphoma of the mucosa-associated lymphoid tissue (MALT) was introduced by Isaacson and Wright in the early 1980s as a distinct clinicopathologic entity with characteristic histologic features (Isaacson and Wright, 1983), and has been incorporated into the REAL-classification under the term ‘extranodal marginal zone B-cell lymphoma of MALT-type’ (Harris et al, 1994). MALT-type lymphomas usually arise in the background of chronic antigenic stimulation triggered by persistent infections and/or autoimmune processes (Greiner et al, 1994). The majority of these tumours occur in the stomach, but this type of lymphoma may affect virtually every organ in the human body, including the intestine, salivary glands, thyroid, lung and ocular adnexa, but also, though less frequently, the skin, urinary bladder and the gonads (Isaacson and Norton 1994; Zucca et al, 1997). Accordingly, thorough staging of patients before initiation of therapy is mandatory once MALT-type lymphoma has been diagnosed, and pretherapeutic work-up should include ophthalmologic examination, otorhinolaryngologic investigation including sonography of the salivary glands or magnetic resonance imaging if indicated, gastroscopy with multiple biopsies, endosonography of the upper GI tract, enteroclysis, colonoscopy, computed tomography (CT) of thorax and abdomen and a bone marrow biopsy. With this staging routine, a relatively high rate of multiorgan involvement could recently be demonstrated in an unselected group of patients with

*Received 26 March 2001*
*Revised 17 July 2001*
*Accepted 20 July 2001*

*Correspondence to:* M Raderer

MALT-type lymphoma of different localizations (Raderer et al, 2000).

According to the finding that malignant lymphomas express receptors (R) for somatostatin (SST), pilot studies using SST analogues as therapeutic agents have been performed (Witzig et al, 1995), and radiolabelled octreotide (OCT), a long-acting SST analogue preferentially binding to SSTR-subtypes 2 and 5, has been used for imaging of such tumours (Goldsmith et al, 1995; Leners et al, 1996; van-den-Anker Lugdenburg et al, 1996), allowing for estimation of the tumour burden after a single tracer injection. In a pilot series performed at our institution (Raderer et al, 1999) using $^{111}$In-DTPA-D-Phe$^{1}$-OCT ($^{111}$In-OCT, OCtreoScan®, Mallinckrodt Medical, St Louis, USA), we could demonstrate a difference in terms of SSTR-expression in gastric versus extragastric MALT-type lymphomas. While no positive scans were obtained in patients with gastric MALT-type lymphomas irrespective of size and stage of the disease, excellent visualization of lymphomas originating in extragastric sites could be achieved using $^{111}$In-OCT. Additional in vitro investigations by means of Northern blotting disclosed expression of mRNA for SSTR2 in tissue samples from 2 patients with extragastric lymphoma, while this SSTR was absent from gastric lymphoma specimens (Raderer et al, 1999).

In view of these findings, we have initiated a follow-up study to investigate prospectively the clinical potential of SSTR scintigraphy using $^{111}$In-OCT for staging and follow-up of patients with extragastric MALT-type lymphoma. In addition, we have also included patients with relapse in or spread to an extragastric site following gastric MALT-type lymphoma to further test the thesis that the site of origin is predictive of in-vivo expression of SSTR with binding affinity for $^{111}$In-OCT.

### PATIENTS AND METHODS

### Patients

Patients with a histologically verified diagnosis of MALT-type lymphoma presenting in an extragastric site as assessed by a reference pathologist (AC) were eligible for this prospective study. Histologic diagnosis of extranodal marginal zone B-cell lymphoma of MALT-type was performed according to the criteria outlined by Isaacson (Isaacson and Wright, 1983) and adopted in the REAL classification (Harris et al, 1994). In addition, immunologic phenotyping on paraffin sections was done for demonstration of light-chain restriction and the phenotype CD20+CD5–CD10-cyclinD1- which, in context with the microscopic appearance, is consistent with low-grade B-cell lymphoma of MALT-type (Harris et al, 1994).

All patients underwent uniform staging procedures before SSTR scintigraphy, consisting of ophthalmologic examination, otorhinolaryngologic investigation including sonography of the salivary glands or magnetic resonance imaging if indicated, gastroscopy with multiple biopsies, endosonography of the upper GI tract, enteroclysis, colonoscopy and bone marrow biopsy. All lesions rated suggestive for the presence of lymphoma were subjected to biopsy, and histologic samples obtained were evaluated by a single individual (AC).

### Somatostatin receptor scintigraphy

Somatostatin-receptor scanning was performed using commercially available $^{111}$In-DTPA-D-Phe$^{1}$-Octreotide (OctreoScan®, Mallinckrodt Medical, St. Louis) 10 μg DTPA-D-Phe$^{1}$-OCT were labelled with 120–150 MBq $^{111}InCl_3$, according to the manufacturer's description, and given by intravenous bolus injection.

For planar and single photon emission tomography (SPET) studies, a large field-of-view gamma camera (Toshiba, Japan) equipped with a medium-energy general-purpose collimator was used. At the time of injection, the field of view covered the abdomen and some of the thorax. For recording and visualization, standard techniques were applied. Sequential images were recorded every minute, starting immediately after the injection for 30 minutes (matrix 128 × 128 pixels). Planar images in anterior, posterior and lateral views were acquired at 30 minutes, between 3 and 6 hours, between 18 and 24 hours and at approximately 72 hours after intravenous injection covering brain/neck, thorax and abdomen (matrix 128 × 128 pixels, 150–300 kcounts, scanning time 10–20 min). SPET acquisition was performed in all patients between 18 and 24 hours after injection. Both energy peaks were used for scanning (set at 173 keV and 247 keV) with a 20% window. SPET imaging was done in a 360° circle, using 6° steps, 30 seconds per step. After processing by a dedicated computer (prefiltering with a Wiener filter, postfiltering with a ramp filter), the data were reconstructed in 3 planes (slice thickness of 7 mm). A yes-or-no system was used to evaluate the presence of neoplastic lesions as judged both by planar and SPET-reconstructions.

### RESULTS

A total of 30 consecutive patients with a diagnosis of MALT-type lymphoma a priori presenting in an extragastric site (none of whom had been included in our previously published series) were studied prospectively (Table 1).

In one patient with disease presentation in the lung, a history of previous gastric MALT-type lymphoma could be established, while staging work-up disclosed secondary involvement of the parotid, the colon, the lacrimal gland and the breast in an additional 4 patients with gastric MALT-type lymphoma. In all 4 patients, HP-associated gastric MALT-type lymphoma had been diagnosed between 9–14 months prior. In 24 patients, no evidence of gastric involvement could be documented: 2 patients presented with lymphoma located in the lung, 9 had lymphoma of the ocular adnexa, 7 had lymphoma in the parotid, 2 patients had disease located in the breast, 3 patients had lymph-node relapse (2 following resection of a MALT-type lymphoma of the thyroid and the parotid, respectively, and one following potentially curative radiation of a lacrimal lymphoma) and one patient had primary lymphoma of the liver.

In addition, also a patient with secondary spread to the stomach, lung and hilar lymph nodes 3 years after radiation of a parotid MALT-lymphoma was included in the series. Multiorgan disease was found in a further 8 patients, including 5 patients with lymphoma of the ocular adnexa with concomitant spread to the pharynx, parotid, lung and breast and bilateral lacrimal lymphoma, while 3 patients had bilateral parotid lymphoma.

All patients underwent scintigraphy with $^{111}$In-OCT before initiation of radiation or chemotherapy, while 13 patients were imaged repeatedly during and/or after completion of therapy. Excellent correlation with conventional imaging results was found for pretherapeutic scans, as all sites of radiologically and histologically verified lymphomatous involvement could be imaged by means of $^{111}$In-OCT. In terms of imaging time, it was found that the early images did not improve the diagnostic capability of the

*© 2001 Cancer Research Campaign*

tracer, and the best results were seen on images obtained 24–48 hours after injection of the tracer. While the tracer is avidly taken up by the spleen, as has repeatedly been published, physiological background due to hepatic or intestinal activity was not a problem in our series, as also lesions within the liver as well as intra-abdominal lymph nodes could be visualized.

In one patient with bilateral parotid lymphoma visualized by means of $^{111}$In-OCT, additional focal tracer uptake in projection to the right maxillary sinus was seen, where the patient had been operated due to chronic sinusitis 6 months prior. Panendoscopy with multiple biopsies revealed the presence of an inflammatory process, but no evidence of lymphoma.

13 patients underwent a second injection of the tracer after completion of therapy, while one patient with MALT-type lymphoma of the liver was scanned after 2 cycles of chemotherapy with 2-CdA and again after 4 cycles of treatment. In this patient, only a slight change in appearance of the liver manifestations was noted on CT-scanning after 2 cycles of therapy, while $^{111}$In-OCT showed markedly decreased tracer uptake, indicating response to chemotherapy. After completion of chemotherapy consisting of 4 cycles of 2-CdA, disappearance of 2 out of 4 initially demonstrated lesions was found on CT and MRI, while scintigraphy still demonstrated tracer uptake in all sites, albeit at reduced intensity, rated suggestive of persistance of lymphoma. The patient underwent surgery with removal of the liver segments initially involved, and histologic work-up disclosed the presence of post-therapeutic fibrosis along with multiple persisting foci of viable lymphoma corresponding to $^{111}$In-OCT scintigraphy. 4 months after surgery, MRI imaging and $^{111}$In-OCT scintigraphy were again performed, showing no indication of persistent lymphoma.

Post-therapeutic scan results showed good correlation with conventional radiologic staging as judged after a median follow up time of 24 months (range; 9–34 months), indicating complete remission following treatment in 7 patients and persistence of

**Table 1** Patient characteristics

| Sex/Age | Localisation | Scan result: Before | Scan result: After therapy | Therapy |
|---|---|---|---|---|
| 1. F/76 | Lung | pos | n.d. | Resection |
| 2. M/63 | Conjunctiva | pos | neg | Radiation |
| 3. F/83 | Conjunctiva | pos | n.d. | Radiation |
| 4. F/78 | Cervical LNN[c] | pos | n.d. | Cyclophosphamide |
| 5. M/59 | Lung[a] | neg | n.d. | CHOP[c] |
| 6. F/49 | Breast | pos | n.d. | Resection |
| 7. M/36 | Lung | pos | n.d. | Resection, CHOP |
| 8. M/76 | Lacrimal (bilateral) | pos | neg | 2CdA[d] |
| 9. M/70 | Stomach | neg | n.d. | Cyclophosphamide |
| | Colon | neg | n.d. | |
| 10. F/69 | Parotid (bilateral) | pos | neg | Radiation |
| 11. M/86 | Lacrimal | pos | pos | 2CdA |
| | Lung | pos | neg | |
| 12. M/69 | Stomach | neg | n.d. | 2CdA |
| | Lung | neg | n.d. | |
| 13. F/37 | Parotid | pos | n.d. | Radiation |
| | Lacrimal | pos | n.d. | Radiation |
| 14. F/51 | Parotid | pos | neg | Resection |
| 15. F/64 | Lacrimal | pos | pos | Radiation |
| 16. F/61 | Stomach | neg | n.d. | Cyclophosphamide |
| | Lacrimal | neg | n.d. | |
| 17. M/32 | Parotid (bilateral) | pos | pos | 2CdA |
| 18. F/71 | Parotid | pos | neg | Radiation |
| 19. F/77 | Lacrimal | pos | n.d. | Radiation |
| 20. F/74 | Abdominal LNN[b] | pos | pos | 2CdA |
| 21. M/38 | Parotid | pos | neg | Radiation |
| 22. F/55 | Breast | pos | n.d. | Resection, radiation |
| 23. F/72 | Lacrimal | pos | neg | Radiation |
| 24. M/63 | Lacrimal | pos | n.d. | Radiation |
| 25. F/67 | Stomach | neg | n.d. | 2CdA |
| | Parotid | neg | n.d. | |
| 26. F/59 | Liver | pos | pos | 2CdA |
| | | | neg | → resection |
| 27. M/74 | Stomach | neg | n.d. | CHOP |
| | Breast | neg | n.d. | |
| 28. M/46 | Parotid | pos | n.d. | Radiation |
| 29. F/69 | Abdominal LNN[f] | pos | n.d. | Radiation |
| 30. F/76 | Stomach[g] | pos | n.d. | CD-20 antibody |
| | Hilar lymph nodes[g] | | | |
| | Lung[g] | | | |

[a]resection of gastric primary, relapse within the lung 6 years later, [b]two years after irradiation of a lacrimal MALT-lymphoma, [c]relapse following two years after resection of a parotid MALT-lymphoma, [d]CHOP = Cyclophosphamide, doxorubicin, vincrstine, prednisone, [e]2CdA = 2-chlorodeoxyadenosine, [f]five years after resection of a thyroid MALT-lymphoma, [g]three years after successful irradiation of a parotid lymphoma.

 *© 2001 Cancer Research Campaign*

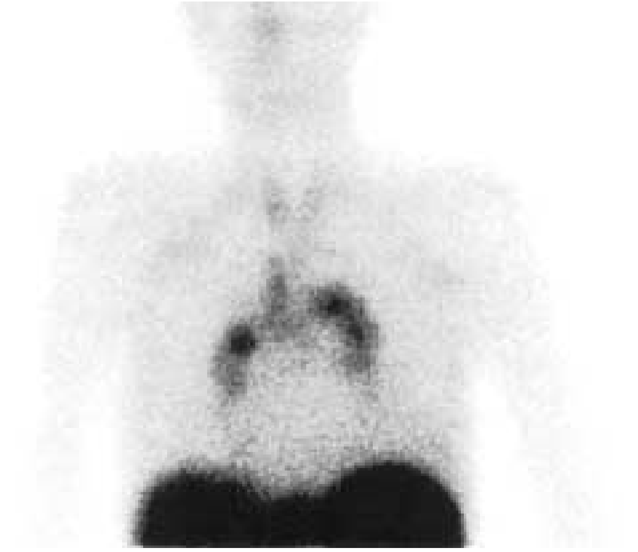

**Figure 1** Planar imaging depicting mediastinal and hilar lymph node relapse following successful irradiation of a parotid MALT-type lymphoma, indicated by multiple hot-spots within the thorax disease in 2 patients. In 3 patients, however, conflicting results were found. In 2 patients with lymphoma of the lacrimal gland, persistent tracer uptake was seen after radiation and chemotherapy (Figure 1) in one patient each, while both ultrasound and magnetic resonance imaging indicated complete remission of the lymphoma. During follow-up, relapse of the disease at the site of tracer accumulation was subsequently seen also on MRI and ultrasound 6 and 9 months later, and both relapses were verified by means of biopsy and histologic work-up. In the third patient, who suffered from bilateral parotid lymphoma, decreased uptake of $^{111}$In-OCT corresponded to partial remission after 4 cycles of chemotherapy as also documented on MRI with subsequent biopsy, but an additional hot spot not present in the pretherapeutic scan was found in projection to the ethmoidal sinus. The correlating MRI image showed the presence of a polypomatous lesion, which was removed in toto and found to be hyperplastic.

In the patient with secondary spread to the stomach, the lung and hilar lymph nodes, focal tracer uptake could be demonstrated, including the site of gastric relapse. As opposed to the excellent scan results obtained in patients with extragastric MALT-type lymphoma, none of the histologically verified lesions could be visualized in the patients with a history of prior gastric lymphoma, irrespective of size or localization.

### DISCUSSION

MALT-type lymphoma is a relatively rare disease, but nevertheless represents the third most common type of lymphoma accounting for about 7% of all non-Hodgkin's lymphomas (Pileri et al, 1998). Both gastric as well as extragastric MALT lymphomas are relatively indolent diseases, which are thought to remain localized for a prolonged period of time. Recent studies, however, have shown that roughly one third of MALT-type lymphomas are disseminated upon diagnosis (Raderer et al, 2000; Thieblemont et al, 2000), with patients suffering from primary extragastric lymphoma being at higher risk for multiorgan involvement (Raderer et al, 2000). In view of this, an exact diagnostic work-up is necessary in order to identify patients suitable for local therapy such as radiation, and diagnostic procedures should be quite extensive according to the pronounced homing tendency of lymphocytes originating from MALT. Thus, methods with the potential to facilitate staging are welcome and warrant clinical evaluation in such patients.

To date, scintigraphic methods have not widely been tested in patients suffering from extranodal B-cell lymphomas of MALT-type. Application of 18F-fluoro-deoxy-glucose positron emission tomography (18F-FDG-PET) has been used for imaging and assessment of viability in various types of lymphoma including Hodgkin's disease. A series performed at our institution, however, has shown that 18F-FDG is not able to visualize sites involved with MALT-type lymphoma, which is probably due to the low proliferative activity of the lymphoma cells (Hoffmann et al, 1999). Recently, we have reported different expression of SSTR in extranodal lymphomas of MALT-type with regard to the origin of the neoplasia. In our pilot study, only patients with primary extragastric MALT lymphomas were found to have positive scans using the radiolabelled SST analogue $^{111}$In-OCT, while visualization of the tumours was not possible in patients with primary gastric MALT-type lymphomas (Raderer et al, 1999). According to these findings in a relatively limited series of patients, we have performed a consecutive study to evaluate the impact of SSTR scintigraphy on staging and follow-up of patients with extragastric MALT lymphoma.

The results of this study demonstrate that SSTR-receptor scintigraphy is a valuable tool for staging and follow-up of such patients. Positive scan results were obtained at the sites of radiologically and histologically ascertained tumoural involvement in all patients with primary extragastric MALT lymphoma. In addition to the excellent accuracy for pretherapeutic staging, non-invasive assessment of therapeutic efficacy was possible in the 13 patients undergoing repeated scanning with $^{111}$In-OCT as judged by a median follow-up period of 24 months (range, 9–34 months). 2 patients, who showed persistence of tracer uptake despite normal conventional work-up following treatment were diagnosed with clinically manifest relapse 6 and 9 months after the respective scintigraphy, and $^{111}$In-OCT was more accurate in determining post-therapeutic residual lymphoma than conventional imaging including CT and MRI in another patient with MALT-type lymphoma of the liver.

In contrast, none of the patients with a negative scintigraphy after therapy has relapsed during the follow-up period in spite of discordant CT results in 2 patients. In these patients, persistent or relapsing lymphoma was suggested on CT scan of the thorax, while no corresponding focal tracer uptake was seen on SSTR scanning. In both patients, persistence of lymphoma was ruled out by unchanged CT over a period of 8 and 14 months, respectively, while fine needle biopsy without evidence of lymphoma was performed in one patient.

In order to further test our hypothesis that gastric MALT lymphomas do not express relevant amounts of SSTR, we have also included 5 patients with extragastric relapse or consecutive dissemination following diagnosis of gastric MALT-lymphoma. In keeping with our initial results, no tracer uptake indicating neoplastic involvement could be seen in these patients. This finding adds further support to the notion that SSTR expression is dependent on the origin of the lymphoma rather than the localization of the lesion. However, a note of caution has to be added, as evaluation of SSTR expression on the cellular level has only been performed in a small pilot series by means of Northern blotting (Raderer et al, 1999) on fresh tumour samples both from gastric as

© 2001 Cancer Research Campaign well as extragastric origin. While the results obtained also indicated a difference in expression of SSTR subtypes, large-scale studies with paraffin-embedded material have not been performed, as there are no commercially available antibodies targetting SSTR subtypes 1–5 suitable for this application. While our data are highly suggestive that there is indeed a difference in terms of SSTR expression on the cellular level, definite proof by immunohistochemistry is still lacking.

Taken together, our data indicate that SSTR scintigraphy using $^{111}$In-OCT is an excellent tool for staging and follow-up in patients suffering from primary extragastric MALT-type lymphoma, allowing for non-invasive evaluation of treatment efficacy, which appears to be superior when compared to conventional imaging techniques. In addition, $^{111}$In-OCT scintigraphy appears to allow distinction between gastric versus extragastric origin of the MALT-type lymphoma in patients presenting with lesions located outside the GI tract. In addition, $^{111}$In-OCT scanning might identify patients suitable for therapy with labelled or unlabelled SST analogues.

### REFERENCES

Goldsmith SJ, Macapinlac HA and O'Brien JP (1995) Somatostatin-receptor imaging in lymphoma. *Semin Nucl Med* **25**: 262–271

Greiner A, Marx A, Heesemann J et al (1994) Idiotype idendity in a MALT-type lyphoma and B-cells in *Helicobacter-pylori*-associated chronic gastritis. *Lab Invest* **70**: 572–578

Harris NL, Jaffe ES, Stein H et al (1994) A revised European-American classification of lymphoid neoplasms: A proposal from the International Lymphoma Study Group. *Blood* **84**: 1361–1392

Hoffmann M, Kletter K, Diemling M et al (1999) F18-FDG-PET does not visualize extranodal B-cell lymphoma of the mucosa-associated lymphoid tissue (MALT)-type. *Ann Oncol* **10**: 1185–1189

Isaacson PG and Norton AJ (1994) Mucosa-associated lymphoid tissue (MALT) and the MALT-lymphoma concept. In: Isaacson PG, Norton AJ (eds) *Extranodal Lymphomas*. pp. 5–14. Churchill Livingstone

Isaacson PG and Wright DH (1983) Malignant lymphoma of mucosa-associated lymphoid tissue. A distinctive type of B-cell lymphoma. *Cancer* **52**: 1410–1416

Leners N, Jamar F, Fiasse R et al (1996) Indium-111-pentreotide uptake in endocrine tumors and lymphomas. *J Nucl Med* **37**: 916–922

Pileri S, Milani M, Fraternali-Orcioni G et al (1998) From the REAL-classification to the upcoming WHO-scheme: A step towards universal categorization of lymphoma entities? *Ann Oncol* **9**: 607–612

Raderer M, Valencak J, Pfeffel F et al (1999) Somatostatin receptor expression in primary gastric versus non-gastric extranodal B-cell lymphoma of MALT-type. *J Natl Cancer Inst* **91**: 716–718

Raderer M, Vorbeck F, Formanek M et al (2000) Importance of extensive staging in patients with mucosa associated lymphoid tissue (MALT)-type lymphoma. *Br J Cancer* **83**: 454–457

Thieblemont C, Berger F, Dumontet C et al (2000) Mucosa-associated lymphoid tissue lymphoma is a disseminated disease in one third of 158 patients analyzed. *Blood* **95**: 802–806

van-den-Anker Lugdenburg PJ, Lowenberg B, Lamberts SW et al (1996) The relevance of somatostatin receptor expression in malignant lymphomas. *Metabolism* **45**: 96–97

Witzig TE, Letendre L, Gerstner J et al (1995) Evaluation of somatostatin analog in the treatment of lymphoproliferative disorders: results of a phase II North Central Cancer Treatment Group trial. *J Clin Oncol* **13**: 2012–2015

Zucca E, Roggera E, Bertoni F et al (1997) Primary extranodal non-Hodgkin's lymphomas. Part 1: Gastrointestinal, cutaneous and genitourinary lymphomas. *Ann Oncol* **8**: 727–737

© 2001 Cancer Research Campaign
